# Serotonin Signals Modulate Mushroom Body Output Neurons for Sustaining Water-Reward Long-Term Memory in *Drosophila*

**DOI:** 10.3389/fcell.2021.755574

**Published:** 2021-11-11

**Authors:** Wang-Pao Lee, Meng-Hsuan Chiang, Li-Yun Chang, Wei-Huan Shyu, Tai-Hsiang Chiu, Tsai-Feng Fu, Tony Wu, Chia-Lin Wu

**Affiliations:** ^1^ Graduate Institute of Biomedical Sciences, College of Medicine, Chang Gung University, Taoyuan, Taiwan; ^2^ Department of Applied Chemistry, National Chi Nan University, Nantou, Taiwan; ^3^ Department of Neurology, Chang Gung Memorial Hospital, Linkou, Taiwan; ^4^ Department of Neurology, New Taipei Municipal Tucheng Hospital, Tucheng, Taiwan; ^5^ Department of Neurology, Xiamen Chang Gung Hospital, Xiamen, China; ^6^ Department of Biochemistry, College of Medicine, Chang Gung University, Taoyuan, Taiwan; ^7^ Brain Research Center, National Tsing Hua University, Hsinchu, Taiwan

**Keywords:** mushroom body, serotonin, 5HT receptor, long-term memory, neuronal circuits of the brain, *Drosophila melanogaster*

## Abstract

Memory consolidation is a time-dependent process through which an unstable learned experience is transformed into a stable long-term memory; however, the circuit and molecular mechanisms underlying this process are poorly understood. The *Drosophila* mushroom body (MB) is a huge brain neuropil that plays a crucial role in olfactory memory. The MB neurons can be generally classified into three subsets: γ, αβ, and α′β′. Here, we report that water-reward long-term memory (wLTM) consolidation requires activity from α′β′-related mushroom body output neurons (MBONs) in a specific time window. wLTM consolidation requires neurotransmission in MBON-γ3β′1 during the 0–2 h period after training, and neurotransmission in MBON-α′2 is required during the 2–4 h period after training. Moreover, neurotransmission in MBON-α′1α′3 is required during the 0–4 h period after training. Intriguingly, blocking neurotransmission during consolidation or inhibiting serotonin biosynthesis in serotoninergic dorsal paired medial (DPM) neurons also disrupted the wLTM, suggesting that wLTM consolidation requires serotonin signals from DPM neurons. The GFP Reconstitution Across Synaptic Partners (GRASP) data showed the connectivity between DPM neurons and MBON-γ3β′1, MBON-α′2, and MBON-α′1α′3, and RNAi-mediated silencing of serotonin receptors in MBON-γ3β′1, MBON-α′2, or MBON-α′1α′3 disrupted wLTM. Taken together, our results suggest that serotonin released from DPM neurons modulates neuronal activity in MBON-γ3β′1, MBON-α′2, and MBON-α′1α′3 at specific time windows, which is critical for the consolidation of wLTM in *Drosophila*.

## Introduction

One of the greatest mysteries in the biological field is how unstable labile memory is consolidated into a stable, long-lasting memory. Memory consolidation in mammals initially requires the hippocampus and cortex, but after the memory is consolidated, the hippocampus is no longer required. Hippocampal damage impairs new memory consolidation while leaving the old memories intact, suggesting that unstable labile memory is formed in the hippocampus and transformed into stable long-lasting memory in the cortex (Scoville and Milner, 1957). Systems memory consolidation model suggests that interaction between hippocampal and cortex is critical during and after an experience (Frankland and Bontempi, 2005). Recent study shows that hippocampal and cortical engram cells participate in both early and late stages of memory, and the dominant location of engram moves from the hippocampus to the cortex during systems consolidation (Kitamura et al., 2017).

Long-term memory (LTM), especially appetitive LTM, allows hungry or thirsty animals to behave appropriately when facing life-supporting experienced events again in the future. LTM is thought to transform from short-term and/or intermediate-term memory and requires a “memory consolidation” process to stabilize short-lasting and labile memory into a long-lasting and stable memory (Margulies et al., 2005). A thirsty fruit fly, *Drosophila melanogaster*, can be trained to associate an odor with water reward to form water-reward memories. The motivational state of fruit flies, such as the thirst for water, induces a more efficient, strong, and robust long-lasting memory compared to the shock-punitive aversive memory (Tully et al., 1994; Shyu et al., 2017). One-session training for pairing of an odor with water induces not only water-reward short-term memory in a few minutes to hours, but also a water-reward LTM (wLTM) that can persist for approximately 2 days (Lin et al., 2014; Shyu et al., 2017; Lee et al., 2020). It has been shown that thirsty-dependent water memory is mediated by neuropeptide leucokinin, which is released from a pair of neurons in the fly brain. Leucokinin controls water memory expression by inhibiting two classes of dopaminergic neurons in the fly brain to permit thirsty-dependent water memory expression (Senapati et al., 2019). wLTM formation in *Drosophila* requires the expression of several genes associated with learning and memory within the brain, as observed in mammals (Scharf et al., 2002; Cepeda et al., 2006; Shyu et al., 2017; Lee et al., 2020).

The mushroom body (MB) is traditionally viewed as the olfactory memory center. The MB is composed of three conventional lobes including the αβ, α′β′, and γ lobes, and contains approximately 2,200 Kenyon cells (Lin et al., 2007). The MB can be further compartmentalized into 15 functional compartments based on the innervated fiber of mushroom body output neurons (MBONs) and dopaminergic mushroom body input neurons (DANs). Each compartment of the MB is modulated by its DANs and the information is read out by its MBONs (Aso et al., 2014a). Importantly, the postsynaptic region of the MBON has high plasticity induced by olfactory associative training, suggesting that MBONs serve as a part of the memory processor (Hige et al., 2015). Different MBONs encode distinct intrinsic valences, which can drive the approach, avoidance, or neutral behavior of *Drosophila.* The summarization of valences from MBONs might be the basic playing keys for performing and recording the behavioral output of memory (Aso et al., 2014b). In our previous studies, we identified that a fruit fly simultaneously associates the water-rewarding event from DAN PAM-β′1 and an odor from projection neurons, both of which converge on MB neurons. After memory acquisition, neurotransmission from MB α′β′ neurons is required for wLTM consolidation. Finally, the fly requires neurotransmission from specific subsets of αβ neurons, γ neurons, and their MBONs to recall the wLTM (Shyu et al., 2017; Lee et al., 2020). It remains unclear how wLTM is transferred from α′β′ neurons to αβ neurons and γ neuron subsets. Moreover, the neuronal circuits and molecules involved in this memory process remain unexplored.

Here, we found that neurotransmitter output in three α′β′-related MBONs, including MBON-γ3β′1, MBON-α′2, and MBON-α′1α′3, is specifically required during wLTM consolidation. Moreover, inhibiting serotonin (5-hydroxytryptamine, 5HT) biosynthesis or blocking neurotransmitter output during memory consolidation in dorsal paired medial (DPM) neurons disrupted wLTM. The GFP Reconstitution Across Synaptic Partners (GRASP) data suggest a connection between DPM neurons and MBON-γ3β′1, MBON-α′2, and MBON-α′1α′3. Finally, RNAi-mediated silencing of 5HT receptors in MBON-γ3β′1, MBON-α′2, and MBON-α′1α′3 disrupted wLTM, suggesting that serotonin signals from DPM neurons mediate neurotransmitter outputs in MBON-γ3β′1, MBON-α′2, and MBON-α′1α′3, which is critical for the consolidation of wLTM.

## Results

### Neurotransmitter Output from α′β′-related MBONs during the First 4-h Period after Training Is Necessary for wLTM

**FIGURE 1:**
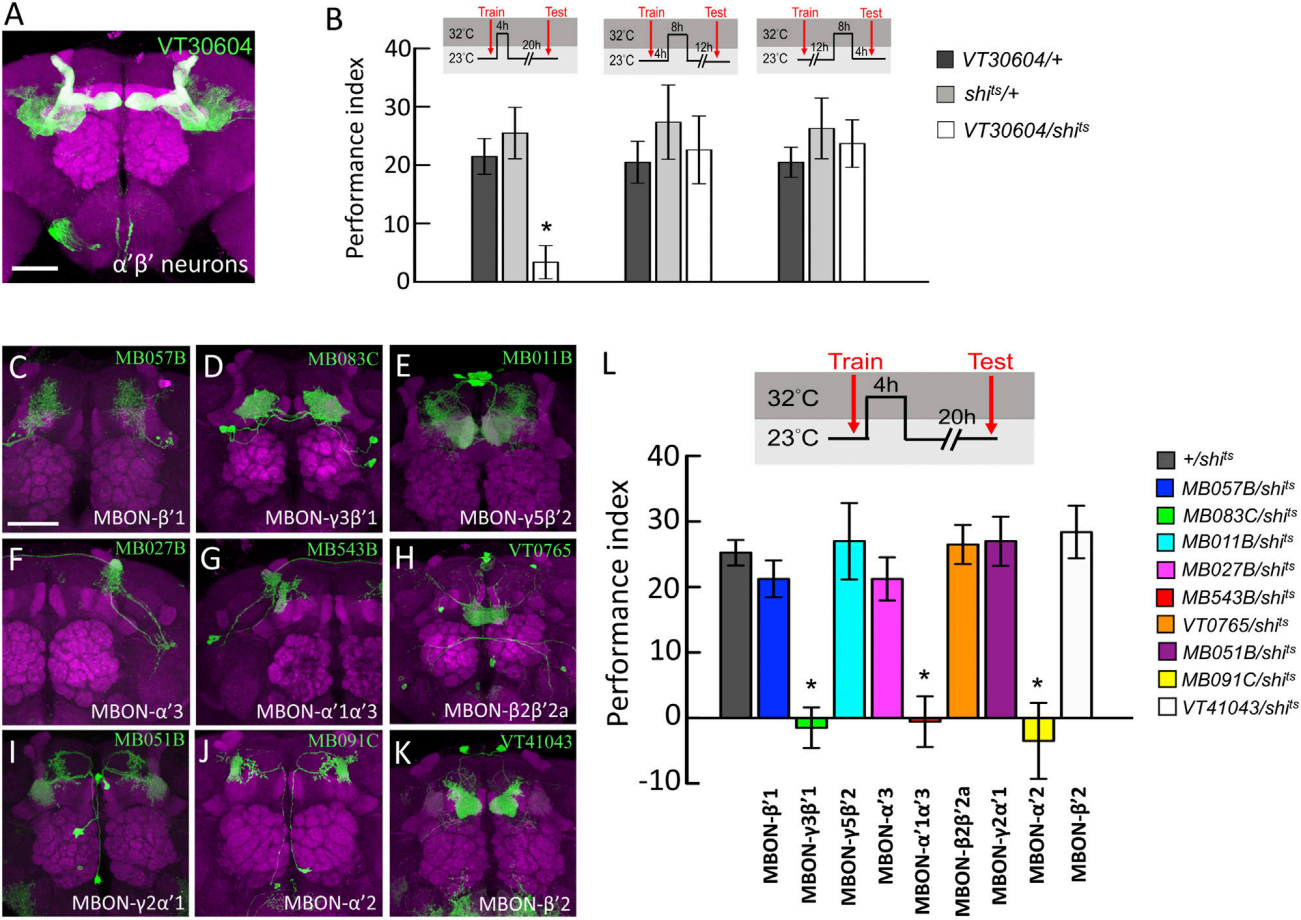
Behavioral screen to identify α′β′-MBONs necessary for wLTM consolidation. **(A)** The expression pattern of *VT30604-GAL4* (green). The brain was immunostained with anti-DLG antibody (magenta). Scale bar, 50 μm. **(B)** Blocking neurotransmitter output in α′β′ neurons at 0–4 h period after training disrupted the wLTM (left panel). Blocking neurotransmitter output in α′β′ neurons at 4–12 h period after training did not affect the wLTM (middle panel). Blocking neurotransmitter output in α′β′ neurons at 12–20 h period after training did not affect the wLTM (right panel). Each value represents mean ± SEM (N = 8, 8, 8, 8, 8, 8, 10, 10, and 10 from left to right bars). ^*^ *p* < 0.05; one-way ANOVA followed by Tukey’s test. **(C)** The expression pattern of *MB057B-GAL4*-driven GFP in MBON-β′1 neurons. **(D)** The expression pattern of *MB083C-GAL4*-driven GFP in MBON-γ3β′1 neurons. **(E)** The expression pattern of *MB011B-GAL4*-driven GFP in MBON-γ5β′2 neurons. **(F)** The expression pattern of *MB027B-GAL4*-driven GFP in MBON-α′3 neurons. **(G)** The expression pattern of *MB543B-GAL4*-driven GFP in MBON-α′1α′3 neurons. **(H)** The expression pattern of *VT0765-GAL4*-driven GFP in MBON-β2β′2a. **(I)** The expression pattern of *MB051B-GAL4*-driven GFP in MBON-γ2α′1, **(J)** The expression pattern of *MB091C-GAL4*-driven GFP in MBON-α′2, and **(K)** The expression pattern of *VT41043-GAL4*-driven GFP in MBON-β′2. All the brains were immunostained with anti-DLG antibody (magenta). Scale bar, 50 μm. **(L)** Blocking neurotransmission in MBON-γ3β′1, MBON-α′1α′3, and MBON-α′2 using *shi* ^*ts*^ during memory consolidation disrupted wLTM. Each value represents mean ± SEM (N = 29, 8, 10, 8, 8, 9, 8, 8, 8, and 10 from left to right bars). ^*^ *p* < 0.05; one-way ANOVA followed by Tukey’s test.

It has been shown that neurotransmitter output from α′β′ neurons is critical for wLTM consolidation but not acquisition or retrieval (Shyu et al., 2017), which led us to ask whether there are time-dependent requirements of neurotransmission in α′β′-related neurons during wLTM consolidation. To address this, we performed acute manipulation of neurotransmitter output by expressing temperature-sensitive *shibire* (*shi* ^*ts*^) allele in specific neurons that inhibits dynamin-mediated endocytosis for recycling of neurotransmitters at restrictive temperatures (Dubnau et al., 2001; Kitamoto, 2001; McGuire et al., 2001). Results showed that blocking neurotransmitter output from α′β′ neurons (via *VT30604-GAL4*) during the first 4-h period right after training disrupts wLTM (Figures 1A,B). However, blocking neurotransmitter output in α′β′ neurons at– 4–12 or 12–20 h after training did not affect wLTM (Figure 1B).

**FIGURE 2:**
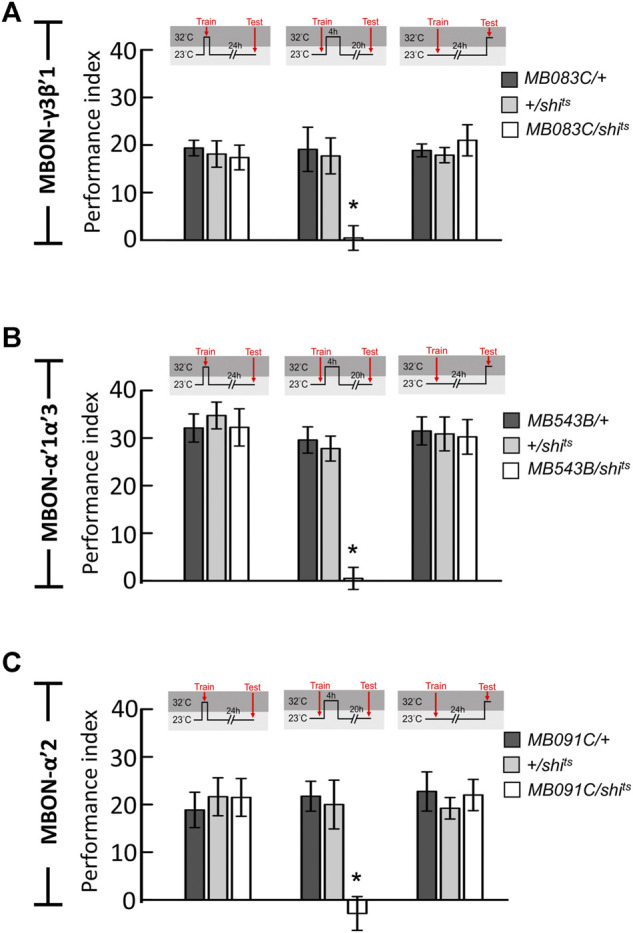
Neurotransmission in MBON-γ3β′1, MBON-α′1α′3, and MBON-α′2 is specifically required for wLTM consolidation but not acquisition or retrieval. **(A)** Blocking neurotransmission in MBON-γ3β′1 (*MB083C-GAL4*) via *shi* ^*ts*^ during memory consolidation, but not during acquisition and retrieval, disrupted wLTM. Each value represents mean ± SEM (N = 8, 9, 8, 11, 14, 13, 8, 8, and 7 from left to right bars). ^*^ *p* < 0.05; one-way ANOVA followed by Tukey’s test. **(B)** Blocking neurotransmission in MBON-α′1α′3 (*MB543B-GAL4*) via *shi* ^*ts*^ during memory consolidation, but not during acquisition and retrieval, disrupted wLTM. Each value represents mean ± SEM (N = 8, 8, 8, 10, 10, 8, 8, 8, and 8 from left to right bars). ^*^ *p* < 0.05; one-way ANOVA followed by Tukey’s test. **(C)** Blocking neurotransmission in MBON-α′2 (*MB091C-GAL4*) via *shi* ^*ts*^ during memory consolidation, but not during acquisition and retrieval, disrupted wLTM (N = 8, 11, 8, 8, 11, 13, 8, 9, and 10 from left to right bars). ^*^ *p* < 0.05; one-way ANOVA followed by Tukey’s test.

Since neurotransmission from α′β′ neurons is specifically required during the early consolidation period (0–4 h after training) of wLTM (Figures 1A,B), we asked whether the α′β′-MBONs are also involved in wLTM consolidation. There are several different types of MBONs, whose dendrites are distributed in different α′β′ lobe subdomains (Aso et al., 2014b). Therefore, we individually tested the involvement of these α′β′-MBONs in wLTM consolidation. MBON-β′1 was labeled using *MB057B-GAL4*, MBON-γ3β′1 was labeled using *MB083C-GAL4*, MBON-γ5β′2 was labeled using *MB011B-GAL4*, MBON-α′3 was labeled using *MB027B-GAL4*, MBON-α′1α′3 was labeled using *MB543B-GAL4*, MBON-β2β′2a was labeled using *VT0765-GAL4*, MBON-γ2α′1 was labeled using *MB051B-GAL4*, MBON-α′2 was labeled using *MB091C-GAL4*, and MBON-β′2 was labeled using *VT41043-GAL4* (Figures 1C–K). Using *shi* ^*ts*^ to block the neurotransmitter output from these α′β′-MBONs individually, we found that blocking neurotransmission only in MBON-γ3β′1, MBON-α′2, or MBON-α′1α′3 during the 4-h period right after training disrupts wLTM (Figure 1L). We further asked whether neurotransmitter output from MBON-γ3β′1, MBON-α′2, and MBON-α′1α′3 is required during training (acquisition) or testing (retrieval). By genetically expressing *shi* ^*ts*^ *,* we found that neurotransmission in MBON-γ3β′1, MBON-α′2, and MBON-α′1α′3 is required only for wLTM consolidation and not for acquisition or retrieval (Figure 2).

### Neurotransmitter Output from MBON-γ3β′1, MBON-α′2, and MBON-α′1α′3 Is Required for wLTM Consolidation at Different Time Periods

**FIGURE 3:**
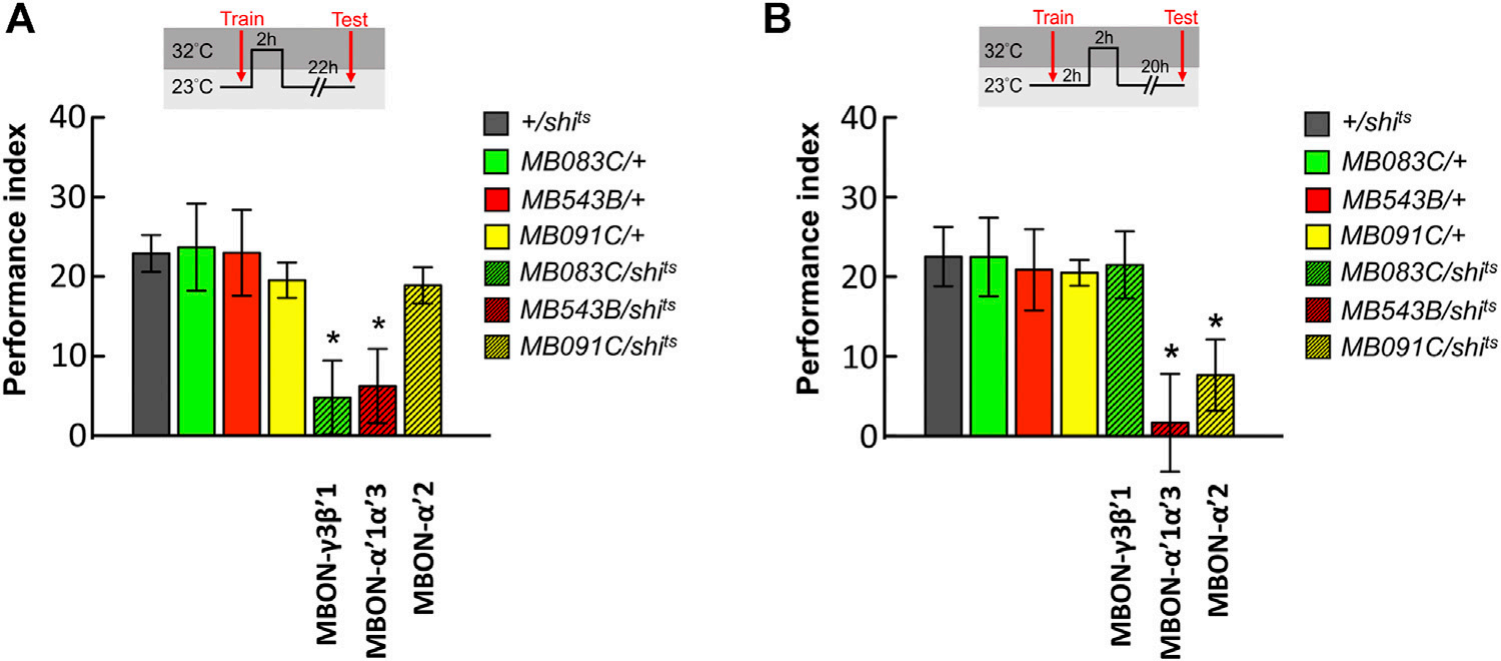
Sequential utilization of α′β′-MBONs for sustaining wLTM consolidation. **(A)** Blocking neurotransmission in MBON-γ3β′1 or MBON-α′1α′3 via *shi* ^*ts*^ at 0–2 h period after training disrupted wLTM. Each value represents mean ± SEM (N = 14, 10, 8, 9, 10, 8, and 9 from left to right bars). ^*^ *p* < 0.05; one-way ANOVA followed by Tukey’s test. **(B)** Blocking neurotransmission in MBON-α′1α′3 or MBON-α′2 via *shi* ^*ts*^ at 2–4 h period after training disrupted wLTM. Each value represents mean ± SEM (N = 13, 12, 9, 8, 12, 9, and 11 from left to right bars). ^*^ *p* < 0.05; one-way ANOVA followed by Tukey’s test.

To dissect the role of MBONs during the initial 4-h period after training, we genetically expressed the *shi* ^*ts*^ transgene in MBON-γ3β′1, MBON-α′2, and MBON-α′1α′3 and performed behavioral assays. All flies were raised and trained at 23°C, maintained at 32°C for 2 h immediately after the training, and shifted back to 23°C for another 22 h and tested. We observed that blocking neurotransmitter output in MBON-γ3β′1 and MBON-α′1α′3 during the 0–2 h period after training disrupted wLTM (Figure 3A). We further investigated the 2–4 h period post-training. The flies were trained at 23°C, maintained at 23°C for 2 h immediately after the training, shifted back to 32°C for 2 h, and again shifted back to 23°C for another 20 h and tested. We observed that blocking neurotransmitter output in MBON-α′2 and MBON-α′1α′3 during the 2–4 h period after training disrupted wLTM (Figure 3B).

### Serotonin Release From DPM Neurons is Necessary for wLTM Consolidation

**FIGURE 4:**
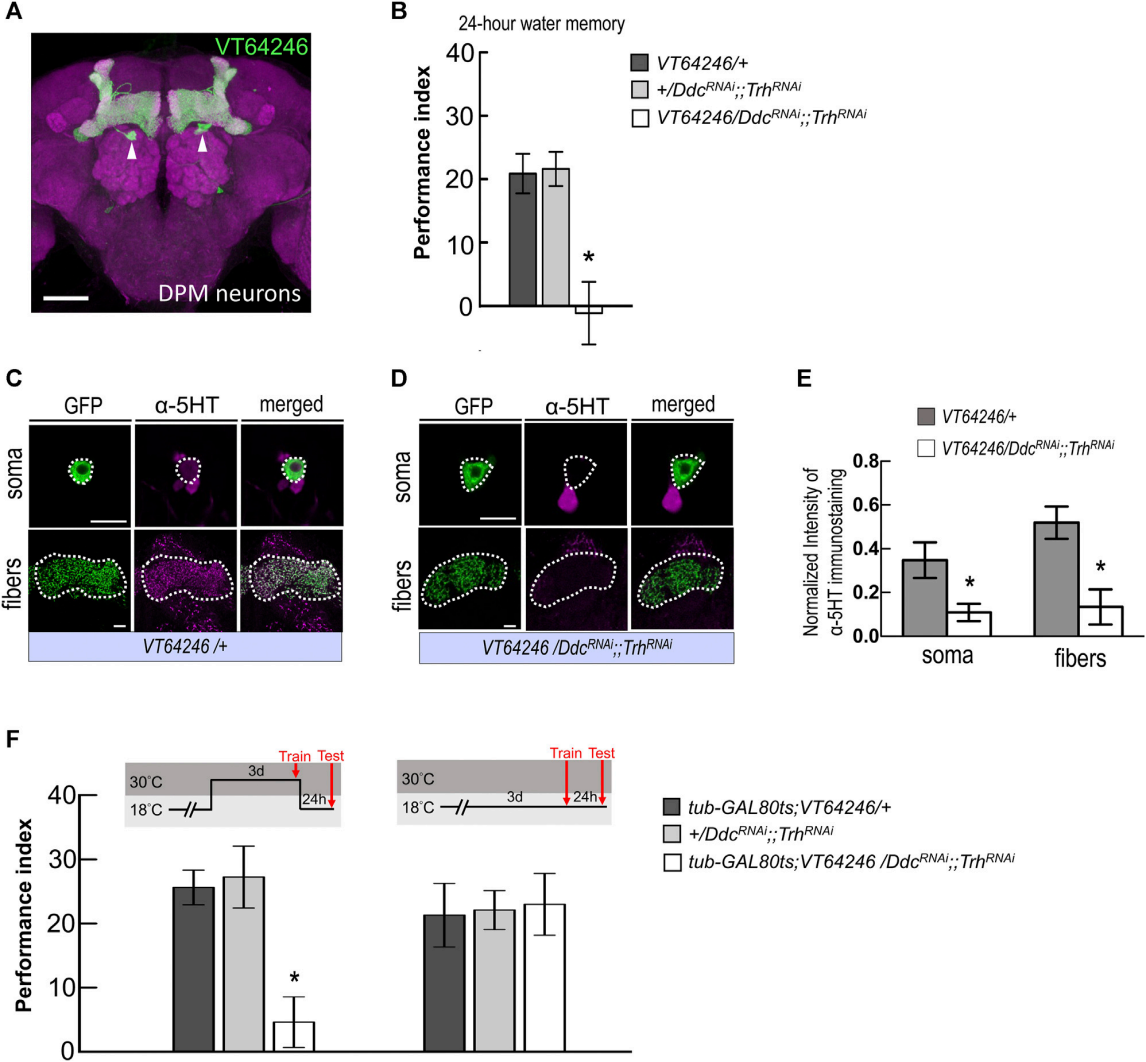
Blocking serotonin biosynthesis in DPM neurons disrupted wLTM. **(A)** The expression pattern of *VT64246-GAL4* (green). White arrowheads indicate the soma of DPM neurons. The brain was immunostained with anti-DLG antibody (magenta). Scale bar, 50 μm. **(B)** Blocking 5HT biosynthesis in DPM neurons by expressing *DDC* ^*RNAi*^ and *Trh* ^*RNAi*^ disrupted the 24-h water memory. Each value represents mean ± SEM (N = 8 for each bar). ^*^ *p* < 0.05; one-way ANOVA followed by Tukey’s test. **(C)** Immunostaining using anti-5HT antibody in DPM neurons and fibers (magenta) in *UAS-mCD8:GFP; VT64246-GAL4/+* flies. Scale bar, 10 μm. Each value represents mean ± SEM (N = 8 and 7 from left to right). ^*^ *p* < 0.05; *t*-test. **(D)** Immunostaining using anti-5HT antibody in DPM neurons and fibers (magenta) in *UAS-mCD8:GFP; VT64246-GAL4 > UAS-Ddc* ^*RNAi*^ *;;UAS-Trh* ^*RNAi*^ flies. Scale bar, 10 μm. **(E)** Quantification of 5HT immunostaining. Single optical section of DPM soma and DPM fibers were analyzed under the same recording condition. The 5HT staining intensity was normalized to the GFP signal. Each value represents mean ± SEM (N = 8, 7, 8, 7 form left to right bars). ^*^ *p* < 0.05; *t*-test. **(F)** Adult-stage-specific knockdown of 5HT biosynthesis in DPM neurons disrupted wLTM (left panel). Each value represents mean ± SEM (N = 8 for each bar). ^*^ *p* < 0.05; one-way ANOVA followed by Tukey’s test. Genetically modified flies showed normal wLTM when kept at 18°C (right panel). Each value represents mean ± SEM (N = 10 for each bar). ^*^ *p* < 0.05; one-way ANOVA.

MBON-γ3β′1, MBON-α′2, and MBON-α′1α′3 receive inputs not only from α′β′ neurons but may also receive inputs from MB modulatory neurons. It has been shown that MB modulatory neurons, the DPM neurons that project to and innervate the MB lobes, play a role in mediating the synaptic activity of MBONs. Previous studies have suggested that DPM neurons are critical for olfactory associative memories, including shock-punishment and sugar-reward conditioning (Waddell et al., 2000; Keene et al., 2004; Yu et al., 2005; Keene et al., 2006; Wu et al., 2011). It has also been reported that DPM neurons release the neurotransmitter 5HT (Lee et al., 2011), which led us to ask whether 5HT synthesis in DPM neurons affects wLTM. In the 5HT biosynthesis pathway, L-tryptophan is first hydroxylated by the enzyme tryptophan hydroxylase (*Trh*) to 5-hydroxytryptophan, which is then decarboxylated by the enzyme dopa decarboxylase (*Ddc*) to 5HT (Blenau and Thamm, 2011). Our results showed that RNAi-mediated silencing of *Trh* and *Ddc* using the GAL4/UAS binary system inhibits 5HT biosynthesis in DPM neurons (via *VT64246-GAL4*) and disrupts wLTM (Figures 4A,B). However, inhibiting 5HT biosynthesis in MB neurons did not affect wLTM (Supplementary Figure S1). We further analyzed the knockdown efficiency of *UAS-Trh* ^*RNAi*^ *; UAS-Ddc* ^*RNAi*^ in *VT64246-GAL4* line. We observed that *VT64246-GAL4 > UAS-Ddc* ^*RNAi*^ *;;UAS-Trh* ^*RNAi*^ flies showed over 50% reduction of anti-5HT antibody immunoreactive signals in both soma and fibers of DPM neurons as compared to the control groups (Figures 4C–E). To exclude the developmental effects of inhibiting 5HT biosynthesis in DPM neurons, we next tested the flies with inducible knockdown of *Ddc* and *Trh* genes using *tub-GAL80* ^*ts*^ in the *VT64246-GAL4* line. Our results showed that adult-stage-specific knockdown of *Ddc* and *Trh* in DPM neurons also impaired wLTM (Figure 4F).

### Neurotransmission in DPM Neurons Is Specifically Required for wLTM Consolidation but Not Acquisition and Retrieval

**FIGURE 5:**
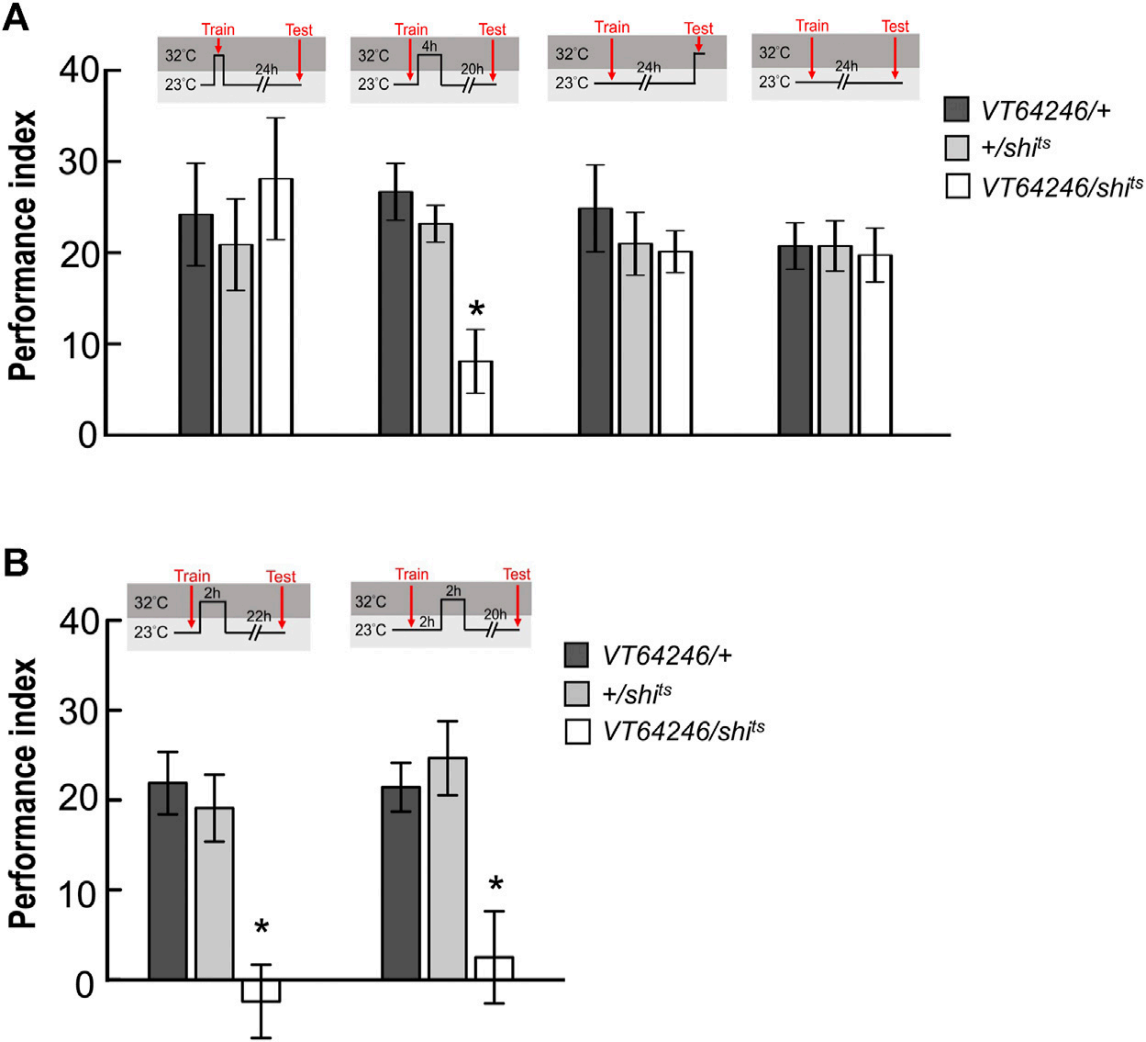
Neurotransmission in DPM neurons is specifically required for wLTM consolidation but not acquisition or retrieval. **(A)** Blocking neurotransmission in DPM neurons via *shi* ^*ts*^ during memory consolidation, but not during acquisition or retrieval, disrupted wLTM. Each value represents mean ± SEM (N = 10, 9, 8, 10, 11, 11, 8, 8, 8, 8, 8, and 8 from left to right bars). ^*^ *p* < 0.05; one-way ANOVA followed by Tukey’s test. **(B)** Blocking neurotransmission in DPM neurons via *shi* ^*ts*^ at 0–2 h period (left panel) or at 2–4 h period (right panel) after training disrupted wLTM. Each value represents mean ± SEM (N = 10, 10, 10, 9, 9, and 8 from left to right bars). ^*^ *p* < 0.05; one-way ANOVA followed by Tukey’s test.

Since wLTM formation requires 5HT biosynthesis in DPM neurons, we asked that neurotransmission in DPM neurons is involved in which memory phases. We genetically expressed *shi* ^*ts*^ transgene in DPM neurons and all transgenic flies were maintained at 23°C and shifted to 32°C to inhibit neurotransmitter output during training (acquisition), trial interval (consolidation), or testing (retrieval). We found that blocking neurotransmission in DPM neurons during consolidation impaired wLTM (Figure 5A). However, blocking neurotransmission in DPM neurons during acquisition or retrieval did not affect wLTM (Figure 5A). Since MBON-γ3β′1, MBON-α′2, and MBON-α′1α′3 showed different time-dependent requirements for wLTM (Figure 3), we asked that in which period during memory consolidation DPM neurons are involved. The flies were trained at 23°C and then maintained at 32°C for 0–2 h or 2–4 h right after the training, and shifted back to 23°C for the resting interval and tested. We found that blocking neurotransmitter output in DPM neurons during the 0–2 h or 2–4 h period disrupted wLTM (Figure 5B). Taken these data together suggest that 5HT neurotransmitter output from DPM neurons is critical for wLTM consolidation (Figures 4, 5).

Considering the gap junctions between DPM and anterior paired lateral (APL) neurons are critical for aversive anesthesia-sensitive memory in *Drosophila* (Wu et al., 2011), which led us to ask whether inhibiting neurotransmission during memory consolidation in APL neurons affects wLTM. Blocking neurotransmission in APL neurons during the 0–4 h period after training did not affect the wLTM, implying that neurotransmission in APL neurons is not critical for wLTM consolidation (Supplementary Figure S2). It is possible that APL neurons are involved in other memory phases rather than wLTM consolidation.

### DPM Neurons Connect to the Dendritic Regions of MBON‐γ3β′1, MBON‐α′2, and MBON-α′1α′3

**FIGURE 6:**
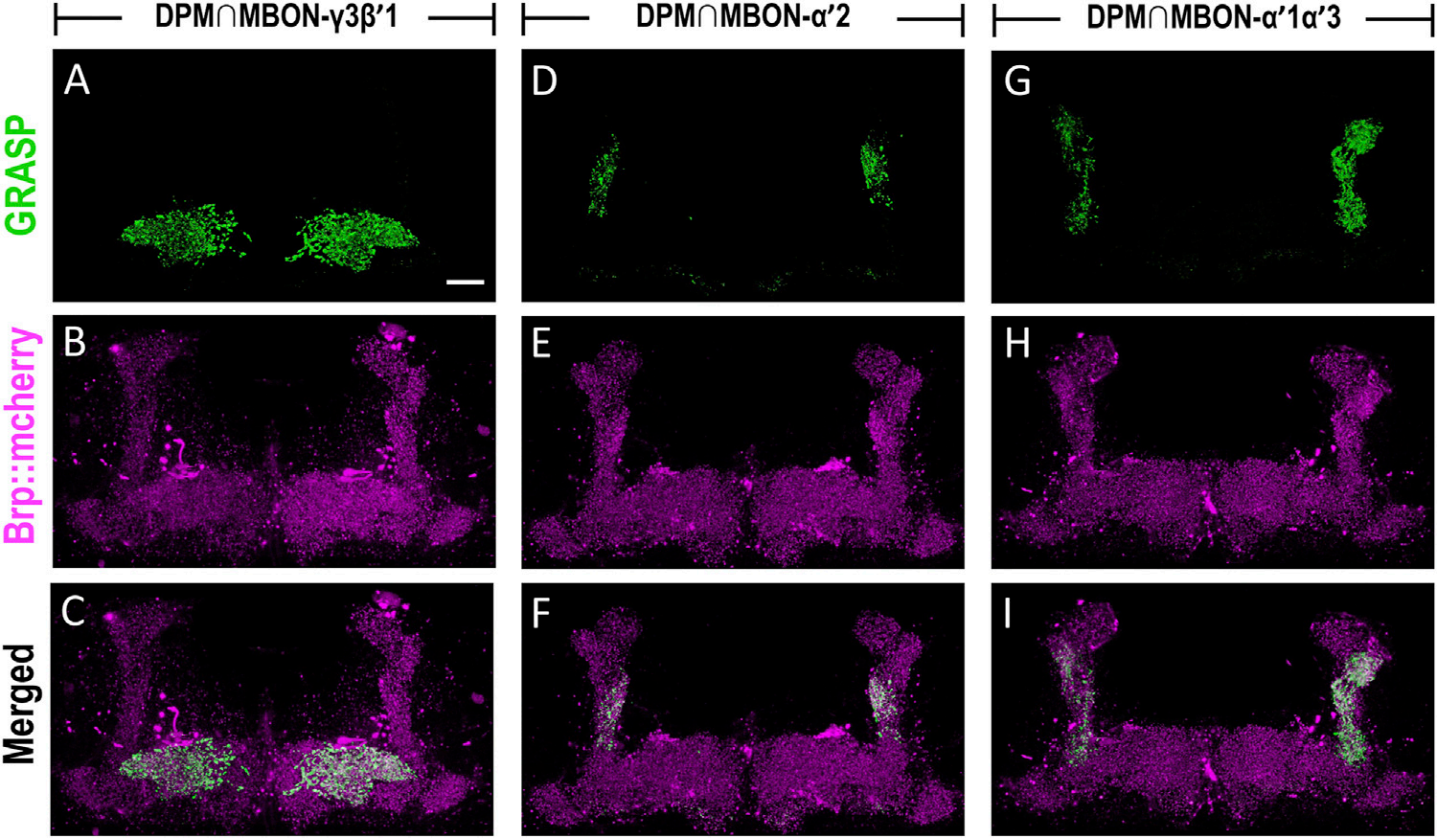
MBON-γ3β′1, MBON-α′2, and MBON-α′1α′3 are downstream circuits of DPM neurons. The connectivity of MBON-γ3β′1, MBON-α′2, MBON-α′1α′3, and DPM were visualized using GRASP. **(A**–**C)** GRASP signals (green) were observed in β′1 and γ3 subregions of MB horizontal lobes in flies carrying *L0111-LexA/+; MB083C-GAL4/lexAop-spGFP*_*11*_ *, UAS-spGFP*_*1-10*_, *lexAop-Brp::mCherry* transgenes. The axons of the DPM neurons were labeled with Brp::mcherry (magenta). **(D**–**F)** GRASP signals (green) were observed in α′2 regions in flies carrying *L0111-LexA/+; MB091C-GAL4/lexAop-spGFP*_*11*_ *, UAS-spGFP*_*1-10*_, *lexAop-Brp::mCherry* transgenes. The axons of the DPM neurons were labeled with Brp::mcherry (magenta). **(G**–**I)** GRASP signals (green) were observed in α′1, α′3, and α′2 subregions in flies carrying *L0111-LexA/+; MB543B-GAL4/lexAop-spGFP*_*11*_ *, UAS-spGFP*_*1-10*_, *lexAop-Brp::mCherry* transgenes. The axons of the DPM neurons were labeled with Brp::mcherry (magenta). Scale bar, 10 μm.

The dendritic regions of MBON-γ3β′1, MBON-α′2, and MBON-α′1α′3 are located in the horizontal and vertical lobes of α′β′ neurons. Considering that axons of DPM neurons are highly innervated into the horizontal and vertical lobes of α′β′ neurons, we investigated the connectivity of DPM neurons with these three MBONs. We utilized the GRASP tool by expressing two split-GFP partners, spGFP_1-10_ and spGFP_11_, to check whether these three types of MBONs are anatomically connected to DPM neurons. Our previous study showed that *L0111-LexA* could label the DPM neurons (Wu et al., 2011) therefore, we chose *L0111-LexA* to drive the expression of *spGFP*_*11*_ and *Brp::mCherry* (labels the axons of DPM neurons). In flies carrying *L0111-LexA > lexAop-spGFP*_*11*_ *, lexAop-Brp::mCherry*, and *MB083C-GAL4 > UAS-spGFP*_*1-10*_, we observed GRASP signals in β′1 and γ3 subregions of MB horizontal lobes (Figures 6A–C). In flies carrying *L0111-LexA > lexAop-spGFP*_*11*_ *, lexAop-Brp::mCherry*, and *MB091C-GAL4 > UAS-spGFP*_*1-10*_, we observed GRASP signals specifically in the α′2 region of the MB vertical lobes (Figures 6D–F). Finally, in flies carrying *L0111-LexA > lexAop-spGFP*_*11*_, *lexAop-Brp::mCherry*, and *MB543B-GAL4 > UAS-spGFP*_*1-10*_, we observed GRASP signals mainly in α′1 and α′3, along with some signals in α′2 subregions of the MB vertical lobes (Figures 6G–I).

### Suppressing 5HT Receptor Expression in MBON-γ3β′1, MBON-α′2, and MBON-α′1α′3 Disrupts wLTM

**FIGURE 7:**
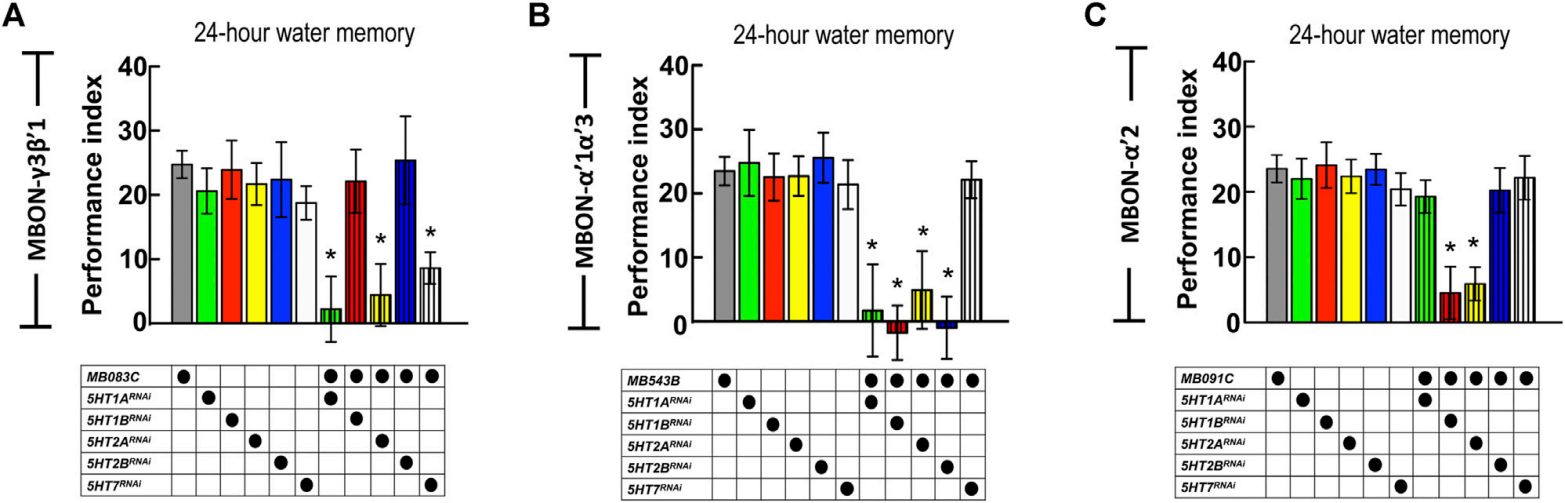
Suppressing 5HT receptor expression in MBON-γ3β′1, MBON-α′1α′3, and MBON-α′2 disrupted wLTM. **(A)** RNAi-mediated silencing of *5HT1A*, *5HT2A*, and *5HT7* in MBON-γ3β′1 disrupted wLTM formation. Each value represents mean ± SEM (N = 26, 10, 10, 10, 8, 12, 10, 9, 10, 8, and 10 from left to right bars). ^*^ *p* < 0.05; one-way ANOVA followed by Tukey’s test. **(B)** RNAi-mediated silencing the *5HT1A*, *5HT1B*, *5HT2A*, and *5HT2B* in MBON-α′1α′3 disrupted wLTM formation. Each value represents mean ± SEM (N = 30, 13, 11, 10, 9, 8, 10, 9, 10, 9, and 8 from left to right bars). ^*^ *p* < 0.05; one-way ANOVA followed by Tukey’s test. **(C)** RNAi-mediated silencing the *5HT1B* and *5HT2A* in MBON-α′2 disrupted wLTM formation. Each value represents mean ± SEM (N = 33, 11, 10, 19, 9, 10, 11, 10, 19, 8, and 12 from left to right bars). ^*^ *p* < 0.05; one-way ANOVA followed by Tukey’s test.

The axons of DPM neurons highly innervate the MB lobe (especially α′β′ lobes), whereas the dendrites of MBON-γ3β′1, MBON-α′2, and MBON-α′1α′3 are located in α′β′ lobes. Our GRASP results showed the connectivity between DPM neurons and the three MBONs. DPM neurons showed overlapping time period requirements with MBON-γ3β′1, MBON-α′2, and MBON-α′1α′3. We asked whether 5HT release from DPM neurons affects MBON-γ3β′1, MBON-α′2, and MBON-α′1α′3 activity during wLTM consolidation. To address this, we suppressed 5HT receptor expression in these three MBONs and assessed wLTM formation. Several 5HT receptor genes have been identified in the *Drosophila* genome including *5HT1A, 5HT1B, 5HT2A, 5HT2B,* and *5HT7* (Majeed et al., 2014). Using the GAL4/UAS binary system, we expressed RNAi against each 5HT receptor gene (Supplementary Figure S3) in MBON-γ3β′1, MBON-α′2, and MBON-α′1α′3, and performed behavioral assays. We found that silencing of *5HT1A*, *5HT2A*, or *5HT7* in MBON-γ3β′1 (via *MB083C-GAL4*) impaired wLTM (Figure 7A). Moreover, silencing of *5HT1A*, *5HT1B*, *5HT2A,* or *5HT2B* in MBON-α′1α′3 (via *MB543B-GAL4*) also impaired wLTM (Figure 7B). Finally, silencing of *5HT1B* or *5HT2A* in MBON-α′2 (via *MB091C-GAL4*) impaired wLTM (Figure 7C). All the memory-impaired flies showed normal odor avoidance and water preference in the thirsty state, suggesting that memory impairment was not caused by the deficiency of sensory inputs (Supplementary Figure S4).

## Discussion

The biogenic amine 5HT modulates several physiological and behavioral responses, such as aggression, feeding, locomotion, sleep, and mood in animals (Weiger, 1997). Several human diseases, including depression, schizophrenia, suicidal behavior, and autism, are linked to serotoninergic system impairment (Jones and Blackburn, 2002). In adult *Drosophila*, several studies have shown that 5HT plays a role in the modulation of behavior including locomotion, aggression, sleep, circadian rhythm, place memory, and aversive memory (Yuan et al., 2005; Yuan et al., 2006; Dierick and Greenspan, 2007; Neckameyer et al., 2007; Sitaraman et al., 2008; Lee et al., 2011). The key finding of our study is that wLTM consolidation requires neurotransmission from three distinct α′β′ output neurons, including MBON-γ3β′1, MBON-α′1α′3, and MBON-α′2, which is mediated by serotonin signals from DPM neurons. This conclusion is supported by four independent lines of evidence. First, blocking neurotransmission during the 0–4 h period after training in MBON-γ3β′1, MBON-α′1α′3, and MBON-α′2 disrupted wLTM (Figures 1–3). Second, inhibiting serotonin biosynthesis or blocking neurotransmission during the 0–4 h period after training in DPM neurons disrupted wLTM (Figures 4, 5). Third, GRASP data suggest that DPM neurons form the upstream circuits of MBON-γ3β′1, MBON-α′1α′3, and MBON-α′2 (Figure 6). Fourth, RNAi-mediated silencing of *5HT1A*, *5HT2A*, and *5HT7* in MBON-γ3β′1 or silencing of *5HT1A*, *5HT1B*, *5HT2A*, and *5HT2B* in MBON-α′1α′3 or silencing of *5HT1B* and *5HT2A* in MBON-α′2 disrupted wLTM (Figure 7).

Neurotransmission in α′β′ neurons is essential for wLTM only during the initial 4 h after training, but not at the later stage during memory consolidation, suggesting that wLTM consolidation is complete within the first 4-h after training. Furthermore, neurotransmission in the three α′β′-related MBONs is required at different periods during consolidation. wLTM formation requires neurotransmission from MBON-γ3β′1 during the 0–2 h period after training, whereas neurotransmission from MBON-α′2 is essential during the 2–4 h period after training. These results suggest the sequential role of MBON-γ3β′1 and MBON-α′2 during wLTM consolidation. In addition, wLTM formation requires neurotransmission from MBON-α′1α′3 during the 0–4 h period after training. Intriguingly, it was shown that MBON-γ3β′1 plays the sleep-promoting role by which activating of MBON-γ3β′1 via *TrpA1* transgene prompts sleep (Aso et al., 2014b). Contrary to this result, a recently study showed that activating MBON-γ3β′1 via *TrpA1* transgene cause the mean sleep amount decrease, whereas inhibiting MBON-γ3β′1 via *shi* ^*ts*^ increase the mean sleep amount (French et al., 2021). Our behavioral result in MBON-γ3β′1 is unlikely caused by sleep loss since blocking MBON-γ3β′1 via *shi* ^*ts*^ did not reduce sleep. Why the different MBONs involved in distinct time period of consolidation remain uncertain. It was shown that dopaminergic MP1 neuronal activity is required for sugar-reward LTM formation, but this activity must be temporally restricted (Pavlowsky et al., 2018). After sugar-reward conditioning, the oscillatory activity in MP1 neurons is enhanced while MVP2 neurons is inhibited. However, in about 30 min after training, the MVP2 neurons are activated and terminate the period of MP1 neuronal activity suggesting that neuronal activity in specific time period is critical for consolidation process (Pavlowsky et al., 2018).

A previous study showed that MBON-γ3β′1 are GABAergic neurons, whereas MBON-α′2 and MBON-α′1α′3 are cholinergic neurons (Aso et al., 2014b) indicating that wLTM consolidation requires one inhibitory GABAergic MBON during the 0–2 h period and two parallel excitatory cholinergic MBONs during 0–2 and 0–4 h periods after training. This implies that both inhibitory and excitatory circuits are involved in the wLTM consolidation process. RNAi-mediated knockdown of glutamate decarboxylase (*GAD*), a rate-limiting enzyme for GABA synthesis, in MBON-γ3β′1 or knockdown of choline acetyltransferase (*ChAT*), a transferase enzyme essential for acetylcholine synthesis, in MBON-α′1α′3 disrupted wLTM (Supplementary Figure S5A,B). Intriguingly, RNAi-medicated knockdown of *ChAT* or knockdown of vesicular acetylcholine transporter (*VAChT*), a transporter responsible for loading of acetylcholine into the synaptic vesicle, in MBON-α′2 did not affect wLTM (Supplementary Figure S5B,C). Therefore, it’s possible that the other type of neurotransmitter releases in MBON-α′2 rather than acetylcholine is critical for wLTM consolidation. It remains unclear why these distinct α′β′-related MBONs act at different periods during wLTM consolidation. We speculate that MBON-γ3α′1, MBON-α′1α′3, and MBON-α′2 are indirectly connected to the γ-dorsal and αβ-surface MB subset neurons, which are required for CREB2- and protein synthesis-dependent wLTM (Lee et al., 2020).

In fruit flies, the 5HT system participates in olfactory learning and memory (Johnson et al., 2011; Sitaraman et al., 2012; Scheunemann et al., 2018; Ganguly et al., 2020), and a previous study suggested that 5HT signals from DPM neurons modulate the activity of the MB αβ neurons, which play a crucial role in olfactory aversive anesthesia-resistant memory formation (Lee et al., 2011). The axons of DPM neurons innervate the MB lobe, in which the MBON dendrites are distributed. Our GRASP data showed the connectivity between DPM neurons and MBON-γ3β′1, MBON-α′1α′3, and MBON-α′2, suggesting that these three MBONs receive inputs from DPM neurons. DPM neurons release the neurotransmitter 5HT and adult-stage-specific inhibition of 5HT biosynthesis in DPM neurons disrupts wLTM. Blocking neurotransmission in DPM neurons during the 0–2 or 2–4 h period after training also disrupts wLTM, suggesting that neurotransmitters from DPM neurons regulate the activity of the downstream MBON-γ3β′1, MBON-α′1α′3, and MBON-α′2. In addition, blocking 5HT biosynthesis in MB neurons by genetic manipulation did not affect wLTM, further confirming that DPM neurons are the source of 5HT rather than MBs.

Hippocampal serotonin receptors participate in the consolidation and reconsolidation of fear conditioning memory in mammals (Schmidt et al., 2017). Fear memory consolidation requires 5HT_5A_, 5HT_6_, and 5HT_7_ receptors in the CA1 region of the hippocampus (Schmidt et al., 2017). In *Drosophila*, there are five different types of 5HT receptors, and our behavioral screening showed that blocking specific 5HT receptors in these three MBONs disrupts wLTM formation. Through genetic manipulation, blocking of 5HT1A, 5HT2A, and 5HT7 in MBON-γ3β′1 or 5HT1A, 5HT1B, 5HT2A, and 5HT2B in MBON-α′1α′3 or 5HT1B and 5HT2A in MBON-α′2 disrupts wLTM. All these genetically manipulated flies showed normal odor acuity and water preference in the thirsty state, which suggests that RNAi-mediated manipulation of 5HT receptor expression in MBONs did not affect sensory inputs. Our results suggest that 5HT release from DPM neurons modulate the activity of MBONs, and this modulation is critical for wLTM consolidation.

In this study, we propose that 5HT release from single paired DPM neurons mediates the neuronal activity of MBON-γ3β′1, MBON-α′2, and MBON-α′1α′3 via different 5HT receptors, and this modulation is critical for sustaining the consolidation of wLTM. Investigating the neuronal circuits that receive inputs from MBON-γ3β′1, MBON-α′2, and MBON-α′1α′3 and identifying the associated GABA and acetylcholine receptors would be an interesting topic for future research.

## Material and Methods

### Fly Stocks

All flies were reared on standard cornmeal food at 25°C and 60% relative humidity on a 12 h:12 h light-dark cycle. *R13F02-GAL4, VT30604-GAL4, VT0765-GAL4, VT41043-GAL4, VT64246-GAL4, APL-GAL4, L0111-LexA, tub-GAL80* ^*ts*^, *UAS-shi* ^*ts*^, and *UAS-mCD8:GFP;UAS-mCD8:GFP* fly strains have been used in our previous studies (Wu et al., 2013; Shih et al., 2015; Wu et al., 2015; Yang et al., 2016; Shyu et al., 2017; Wu et al., 2018; Shyu et al., 2019; Lee et al., 2020). The *lexAop-spGFP*_*11*_ *, UAS-spGFP*_*1-10*_, *lexAop-Brp::mCherry* fly strains were obtained from Tzu-Yang Lin. The *UAS-DDC* ^*RNAi*^ and *UAS-Trh* ^*RNAi*^ fly strains were obtained from Ann-Shyn Chiang (Lee et al., 2011). The *UAS-GAD* ^*RNAi*^ (v32344) fly strain was obtained from Vienna *Drosophila* RNAi Center. *MB057B-GAL4, MB083C-GAL4, MB011B-GAL4, MB027B-GAL4, MB543B-GAL4, MB051B-GAL4, MB091C-GAL4, UAS-5HT1A* ^*RNAi*^ (TRiP.JF01852)*, UAS-5HT1B* ^*RNAi*^ (TRiP.JF01851)*, UAS-5HT2A* ^*RNAi*^ (TRiP.JF02157)*, UAS-5HT2B* ^*RNAi*^ (TRiP.HMJ22882)*, UAS-5HT7* ^*RNAi*^ (TRiP.JF02576), *UAS-ChAT* ^*RNAi*^ (TRiP.JF01877), and *UAS-VAChT* ^*RNAi*^(TRiP.JF02764) fly strains were obtained from the Bloomington *Drosophila* Stock Center.

### Whole-Mount DLG Immunostaining

The fly brains were dissected in phosphate-buffered saline (PBS) solution and immediately transferred to 4% paraformaldehyde (PFA) for fixation for 20 min. Fixed brain samples were incubated in penetration and blocking buffer (PBS containing 2% Triton X-100 and 10% normal goat serum) for 2 h. During the penetration and blocking period, the brain samples were subjected to a degassing procedure. Brains were then incubated in the dilution buffer (PBS containing 0.25% Triton X-100, 1% normal goat serum) containing mouse 4F3 anti-discs large (DLG) monoclonal antibody (1:10, AB 528203, Developmental Studies Hybridoma Bank, University of Iowa) at 25°C for 24 h. After incubation, the tissues were washed in PBS containing 1% Triton X-100 (PBS-T) three times (each time 15 min), and incubated with biotinylated goat anti-mouse IgG (1:200, 31,800, Thermo Fisher Scientific) at 25°C for 24 h. Next, the brain samples were washed and incubated with Alexa Fluor 635 streptavidin (1:500, S32364, Thermo Fisher Scientific) at 25°C for 24 h. After intensive washing in PBS-T for three times, the brain samples were cleared and mounted in FocusClear (FC-101, CelExplorer).

### Quantification of 5HT Immunostaining

Fly brains were immunostained with rabbit anti-5HT polyclonal antibody (1:200, S5545, Sigma-Aldrich) at 25°C for 24 h. After three washes in PBS-T, the samples were incubated in biotinylated goat anti-rabbit IgG (1:200, 31,822, Thermo Fisher Scientific) at 25°C for 1 day. The brain samples were then washed and incubated in Alexa Fluor 635 streptavidin (1:500, S32364, Thermo Fisher Scientific) at 25°C for 1 day. After three-time extensive washing, the brain samples were cleared and mounted in FocusClear (FC-101, CelExplorer). Brain images were obtained using a Zeiss LSM 700 confocal microscope under the same confocal settings for each brain sample, and the images were further analyzed using ImageJ. Single optical sections were used to calculate the average intensity values per voxel of the 5HT immunopositive signals in the soma and fibers of DPM neurons, and the signals were normalized to the GFP intensity.

### Confocal Microscopy

The fly brain samples were imaged under the Zeiss LSM 700 confocal microscope with a ×63 Plan-Apochromat oil-immersion objective lens (N.A. value, 1.4; working distance, 170 μm) or a ×40 C-Apochromat water-immersion objective lens (N.A. value, 1.2; working distance, 220 μm). The confocal pinhole (optical section) was set at 1.5 μm when using ×63 objective lens or 2 μm when imaging thorough ×40 objective lens. The brain image stacks were processed using the ZEN software.

### GRASP Analysis

Adult fly brains carrying *L0111-LexA > lexAop-spGFP*_*11*_ *, lexAop-Brp::mCherry*, and *MB083C-GAL4 > UAS-spGFP*_*1-10*_ were used for DPM and MBON-γ3β′1 connectivity assay. Adult fly brains carrying *L0111-LexA > lexAop-spGFP*_*11*_ *, lexAop-Brp::mCherry*, and *MB091C-GAL4 > UAS-spGFP*_*1-10*_ were used for DPM and MBON-α′2 connectivity assay. Adult fly brains carrying *L0111-LexA > lexAop-spGFP*_*11*_, *lexAop-Brp::mCherry*, and *MB543B-GAL4 > UAS-spGFP*_*1-10*_ were used for DPM and MBON-α′1α′3 connectivity assay. All the brain sample were dissected in PBS solution and immediately transferred to 4% PFA for fixation and degassing procedure. The fixed brain samples were than cleared and mounted in FocusClear and imaged under Zeiss LSM 700 confocal microscope by using 488 and 555 nm lasers for GFP and mCherry excitation respectively.

### Behavioral Assay

Flies were deprived of water by keeping them in a glass milk bottle containing a 6 cm × 3 cm piece of dry sucrose-soaked filter paper (the filter paper was soaked in saturated sucrose solution and allowed to dry before use) at 23°C and 20–30% humidity for 16 h before water conditioning. Approximately 50 water-deprived flies were transferred to the plastic training tube of the T-maze (TM-101, CelExplorer) and provided with a stream of relatively odorless room air for 1 min. The flies were exposed to the first odor for 2 min [unconditioned stimulus, CS–: 3-octanol (OCT) or 4-methylcyclohexanol (MCH)] in a plastic tube lined with dry filter paper, followed by 1 min of room air. The flies were then transferred to another plastic tube containing a water-soaked filter paper and exposed to a second odor for 2 min (conditioned stimulus, CS+: MCH or OCT). Finally, the flies were transferred to a clean training tube and exposed to fresh room air for 1 min. The trained flies were kept in a plastic vial containing a 1.5 cm × 3 cm piece of dried sucrose-soaked filter paper during the 24 h interval between training and testing. In the testing phase, flies were presented with a choice between CS+ and CS–odors in a T-maze for 2 min. The performance index (PI) was calculated as the number of flies running toward the CS + odor minus the number of flies running toward the CS–odor, divided by the total number of trained flies and multiplied by 100. For calculating individual PI, naive flies were first trained by pairing water with OCT (CS+), and the index (PI_O_) was calculated. Next, another group of naive flies was trained by pairing water with MCH (CS+), and the index (PI_M_) was calculated. The individual PI was calculated as the average of the single PI_O_ and PI_M_ values.

### Odor Avoidance and Water Preference Assays

Groups of approximately 50 naive flies were given a choice between either MCH or OCT versus ‘fresh’ room air for 2 min in the T-maze for the odor avoidance assay. The odor avoidance index was calculated as the number of flies in the fresh room air tube minus the number of flies in the MCH or OCT tube, divided by the total number of flies, and multiplied by 100. Groups of approximately 50 naive water-deprived flies were given 2 min to choose between tubes containing a water-soaked filter paper or a dry filter paper in T-maze in the water preference assay. The water preference index was calculated as the number of flies in the tube containing the water-soaked filter paper minus the number of flies in the tube containing the dry filter paper, divided by the total number of flies, and multiplied by 100.

### Quantitative PCR

The efficiency of gene silencing in each 5HT receptor RNAi line from collections was verified with qPCR. Flies for qPCR experiments were generated by crossing virgin *elav-GAL4* flies to either wild-type males or the various male *UAS-5HT receptor* ^*RNAi*^ flies. The RNA from isolated heads of adult flies was extracted by the TRIzol Reagent (T9424, Sigma-Aldrich). The extracted RNA was used to synthesize first strand of cDNA with SuperScript^TM^III First-Strand Synthesis SuperMix Kit (18080400, Thermo Fisher Scientific). The expression levels of mRNA were quantified with SYBR Green PCR Master Mix on a StepOnePlus System.

### Statistical Analysis

All the raw data were analyzed parametrically using the Prism 5.0 software (GraphPad). Comparison between more than two groups was performed using one-way analysis of variance (ANOVA) followed by the Tukey’s multiple comparison test. Comparison between two groups was performed using the paired *t*-test. Statistical significance was set at *p* < 0.05. Data are presented as mean ± standard error of the mean (SEM).

## Data Availability

The original contributions presented in the study are included in the article/Supplementary Material, further inquiries can be directed to the corresponding author.
