# Supplementary material for: Serotonin Signals Modulate Mushroom Body Output Neurons for Sustaining Water-Reward Long-Term Memory in Drosophila
Source: Front Cell Dev Biol. 2021 Nov 11;9:755574. doi: 10.3389/fcell.2021.755574 (PMC8631865; doi:10.3389/fcell.2021.755574)
Supplement: Supplementary file 1 [file DataSheet1.docx]

**Supplementary Information**


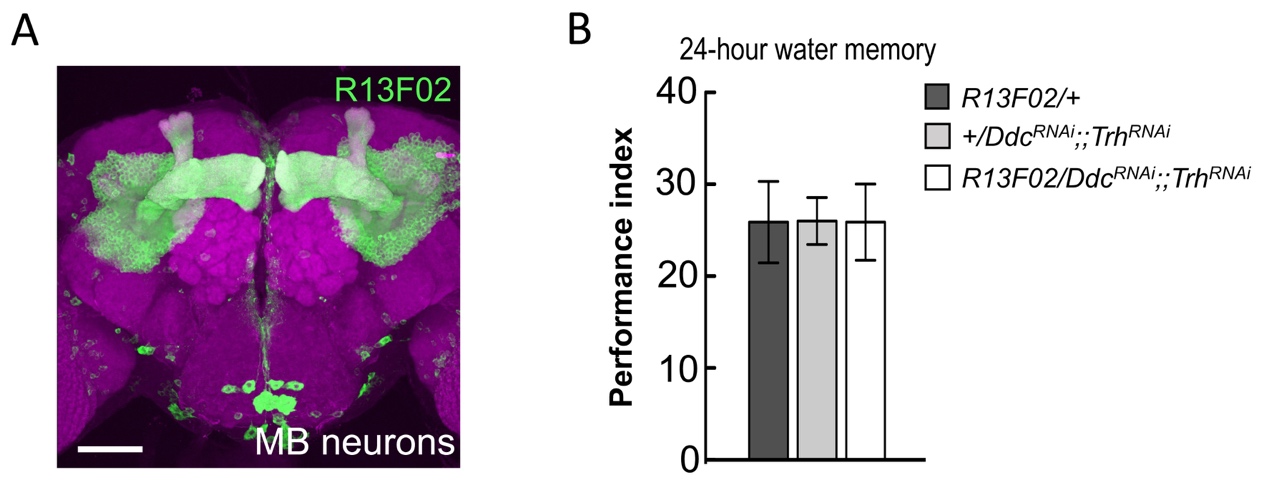


**Supplementary Figure 1. Blocking serotonin biosynthesis in MB neurons did not affect wLTM.**

(**A**) The expression pattern of *R13F02-GAL4* (green). The brain was immunostained with anti-DLG antibody (magenta). Scale bar, 50 μm. (**B**) Blocking 5HT biosynthesis in MB neurons via expression of *Ddc^RNAi^* and *Trh^RNAi^* did not affect the 24-hour water memory. Each value represents mean ± SEM (N = 8 for each bar); p > 0.05; one-way ANOVA.

**
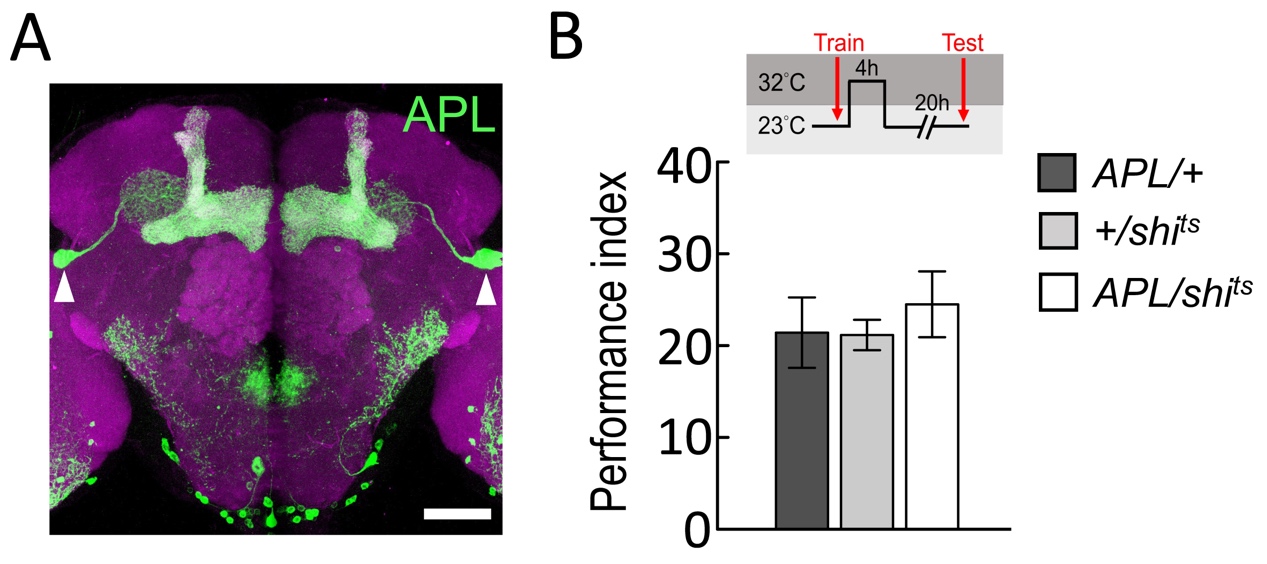
 Supplementary Figure 2. Blocking neurotransmission in APL neurons at 0-4 hour period after training did not affect wLTM.**

(**A**) The expression pattern of *APL-GAL4* (green). White arrowheads indicate the soma of APL neurons. The brain was immunostained with anti-DLG antibody (magenta). Scale bar, 50 μm. (**B**) Blocking neurotransmission in APL neurons via *shi^ts^* at 0-4 hour period after training did not affect wLTM. Each value represents mean ± SEM (N = 12, 13, and 12 from left to right bars). p > 0.05; one-way ANOVA.


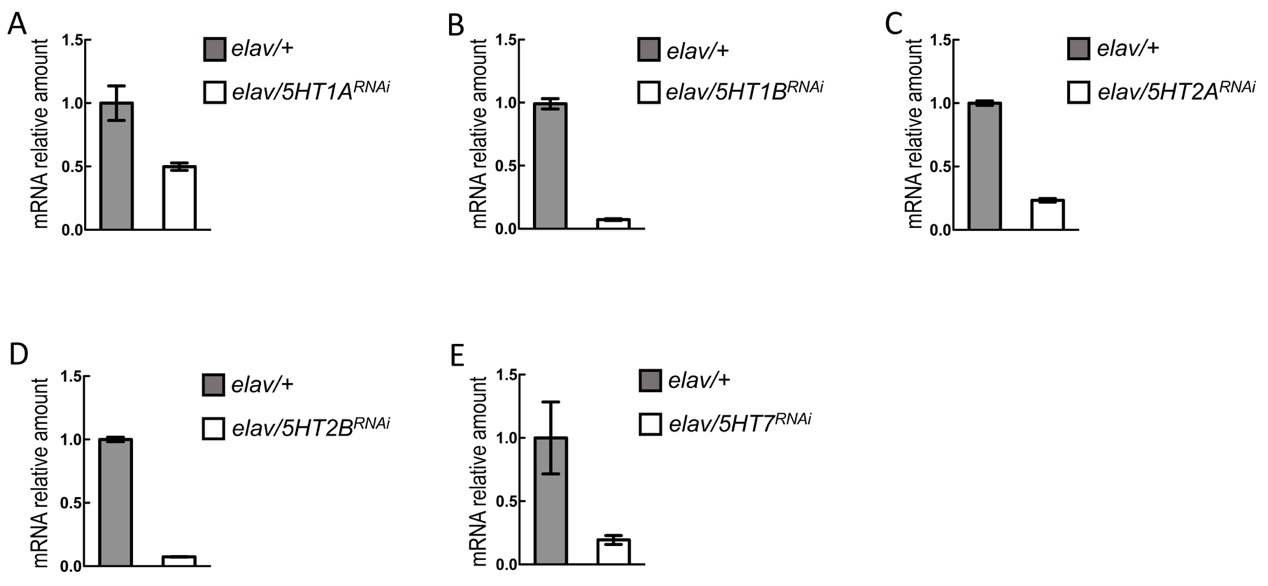


**Supplementary Figure 3. Effectiveness of 5HT receptors RNAi lines used in this study.**

qPCR data showing relative 5HT receptor mRNA levels in manipulated flies (*elav-GAL4/UAS-5HT receptor^RNAi^*) and control flies (*elav-GAL4/+*). 60S ribosomal protein L32 (*RPL32*) was used as internal normalizer. Each value represents mean ± SEM (N = 4 for each bar). The primers used were as follows:

(**A**) anti-5HT1A mRNA (TRIP.JF01852): F, 5’-ATCCCGAACAGCCGAATGTAG-3’

and R, 5’-CCGCTCGTCTTTTACCGGA-3’.
(**B**) anti-5HT1B mRNA (TRiP.JF01851): F, 5’-GGACACGACTAGCAATCTCAGC-3’ and R, 5’-GGCGGCAATTACGAATACGTT-3’.

(**C**) anti-5HT2A mRNA (TRiP.JF02157): F, 5’-ACCCAACAACACATTCCAGTG-3’ and R, 5’-TTTCAGACAATGGGCTTTCCC-3’.

(**D**) anti-5HT2B mRNA (TRiP.HMJ22882): F, 5’-GATCTGCCTGGATGTACTCTTCT-3’ and R, 5’-ATCGCATTGGGTATCGTAGCG-3’.

(**E**) anti-5HT7 mRNA (TRiP.JF02576): F, 5’-CACTTCGCAGGACTTTAATAGCA-3’ and R, 5’-GCCTTGATACGAGACTATGGC-3’.


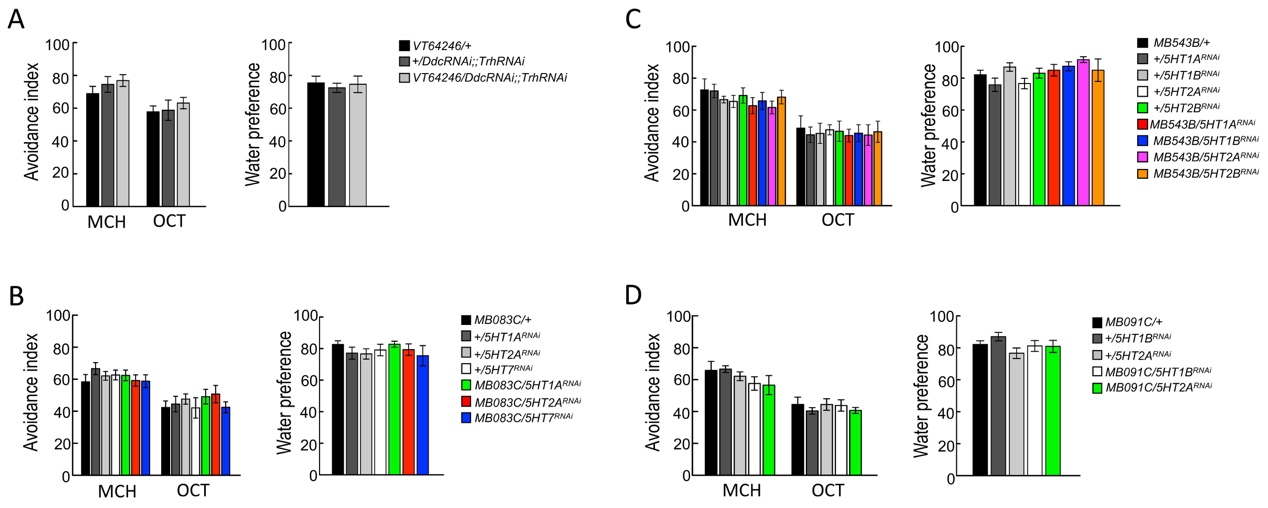


**Supplementary Figure 4. Normal odor avoidance and water preference in genetically manipulated flies used in this study.**

(**A**) Flies carry *VT64246-GAL4/UAS-Ddc^RNAi^;;UAS-Trh^RNAi^* transgenes showed

normal odor avoidance and water preference behavior. Each value represents mean ± SEM (N = 8, 8, and 8 for MCH; N = 9, 9, and 9 for OCT; N=8, 10, and 9 for water preference); p > 0.05; one-way ANOVA. (**B**) Flies carrying *MB083C-GAL4/UAS-5HT1A^RNAi^, MB083C-GAL4/UAS-5HT2A^RNAi^,* or *MB083C-GAL4/UAS-5HT7^RNAi^* transgenes showed normal odor avoidance and water preference. Each value represents mean ± SEM (N = 8, 8, 9, 9, 10, 10, and 10 for MCH; N = 8, 10, 8, 10, 9, 8, and 8 for OCT; N = 8, 8, 8, 9, 8, 9, and 8 for water preference); p > 0.05; one-way ANOVA. (**C**) Flies carrying *MB543B-GAL4/UAS-5HT1A^RNAi^, MB543B-GAL4/UAS-5HT1B^RNAi^, MB543B-GAL4/UAS-5HT2A^RNAi^,* or *MB543B-GAL4/UAS-5HT2B^RNAi^* showed normal odor avoidance and water preference behavior. Each value represents mean ± SEM (N = 8, 8, 9, 9, 8, 9, 8, 10, and 8 for MCH; N = 8, 10, 8, 8, 10, 8, 8, 10, and 9 for OCT; N = 9, 8, 8, 8, 9, 10, 8, 8, and 8 for water preference); p > 0.05; one-way ANOVA. (**D**) Flies carrying *MB091C-GAL4/UAS-5HT1B^RNAi^* or *MB091C-GAL4/UAS-5HT2A^RNAi^* transgenes showed normal odor avoidance and water preference. Each value represents mean ± SEM (N = 9, 9, 9, 9, and 9 for MCH; N = 9, 8, 8, 9, and 9 for OCT; N = 8, 8, 8, 12, and 10 for water preference); p > 0.05; one-way ANOVA.


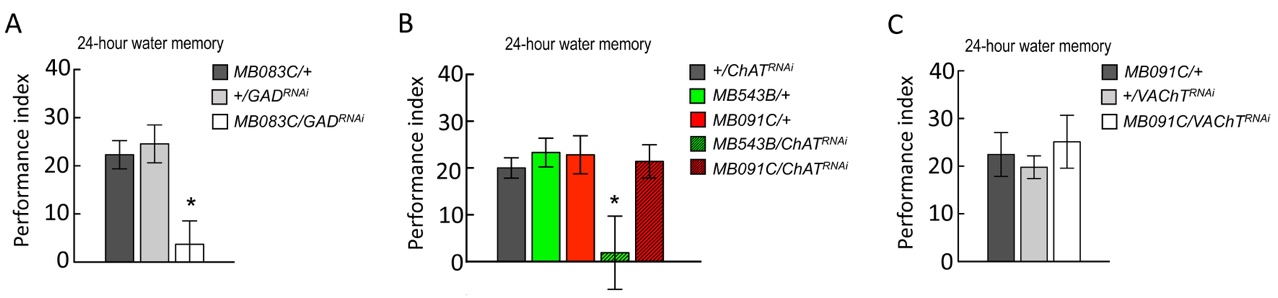


**Supplementary Figure 5. GABA and acetylcholine synthesis in MBONs are critical for wLTM.**

(**A**) Blocking GABA biosynthesis in MBON-γ3β′1 via expression of *GAD^RNAi^* (Flies carry *MB083C-GAL4/UAS-GAD^RNAi^* transgenes) showed 24-hour water memory defect. Each value represents mean ± SEM (N = 10, 9, and 10). *p < 0.05; one-way ANOVA followed by Tukey’s test. (**B**) Blocking acetylcholine biosynthesis in MBON-α′1α′3 via expression of *ChAT^RNAi^* (Flies carry *MB543B-GAL4/UAS-ChAT^RNAi^* transgenes) showed 24-hour water memory defect. Blocking acetylcholine biosynthesis in MBON-α′2 via expression of *ChAT^RNAi^* (Flies carry *MB091C-GAL4/UAS-ChAT^RNAi^* transgenes) did not affect 24-hour water memory. Each value represents mean ± SEM (N = 9, 10, 11, 10 and 10 from left to right bars). *p < 0.05; one-way ANOVA followed by Tukey’s test. (**C**) RNA-mediated silencing of *VChAT* in MBON-α′2 via expression of *VChAT^RNAi^* (Flies carry *MB091C-GAL4/UAS- VChAT^RNAi^* transgenes) did not affect 24-hour water memory. Each value represents mean ± SEM (N = 8 for each bar). p > 0.05; one-way ANOVA.
